# Programmed Death-Ligand 1 (PD-L1) expression and clinical outcomes in oral squamous cell carcinoma: evidence from a large clinical cohort

**DOI:** 10.1007/s10006-026-01628-3

**Published:** 2026-08-29

**Authors:** Michael Alfertshofer, Maximilian Richter, Konrad Klinghammer, Christian Doll, Steffen Koerdt, Max Heiland, Friedrich Mrosk

**Affiliations:** 1 Department of Oral and Maxillofacial Surgery, Charité – Universitätsmedizin Berlin, corporate member of Freie Universität Berlin, and Humboldt-Universität Zu Berlin, Berlin, Germany; 2 Department of Plastic and Hand Surgery, Technical University Munich, Munich, Germany; 3 Division of Hematology, Oncology and Tumorimmunololgy, Charité – Universitätsmedizin Berlin, Comprehensive Cancer Center, corporate member of Freie Universität Berlin, and Humboldt-Universität Zu Berlin, Berlin, Germany

**Keywords:** Head and neck squamous cell carcinoma, PD-L1 expression, Prognostic biomarkers, Overall survival

## Abstract

**Background:**

Despite the recognized biological relevance of the PD-1/PD-L1 axis in immune evasion, the prognostic significance of PD-L1 expression in surgically treated oral squamous cell cancer (OSCC) patients remains largely unclear.

**Objective:**

To investigate the prognostic significance of PD-L1 expression in a large cohort of OSCC patients primarily treated surgically in a single center.

**Methods:**

This analysis included 156 consecutive OSCC patients treated with curative-intent surgery with their clinicopathologic variables, treatment details and clinical treatment outcomes. PD-L1 expression was categorized using the Combined Positive Score (CPS), defining PD-L1 positivity as CPS ≥ 10. Sensitivity analyses were repeated at the alternative thresholds CPS ≥ 1 and CPS ≥ 20. Survival and recurrence endpoints (OS, DFS, LRCR, NCR) were evaluated using Kaplan–Meier/log-rank methods and Cox regression with multivariable adjustment for key confounders.

**Results:**

Of 156 consecutive OSCC patients, 120 (76.9%) were PD-L1-positive. PD-L1 status was not associated with any clinicopathological parameter investigated herein. At 3 years, OS (76.1% vs. 68.7%), DFS (63.5% vs. 42.6%), LRCR (77.1% vs. 75.5%), and NCR (93.0% vs. 91.0%) were comparable between PD-L1-positive and PD-L1-negative patients, respectively. Neither binary PD-L1 classification nor continuous CPS was associated with OS, DFS, LRCR, or NCR in multivariable Cox regression, with no differential effect by disease stage. In adjuvant-treatment-stratified analysis, PD-L1 positivity was associated with improved DFS in patients without adjuvant therapy, but not in those who received it. In sensitivity analyses, findings at CPS ≥ 20 were concordant with the primary analysis, whereas at CPS ≥ 1 PD-L1 positivity was associated with superior OS (HR = 0.280 [95% CI: 0.106–0.745]; *p* = 0.011).

**Conclusion:**

Within a mean follow-up of 15.7 ± 12.5 months, PD-L1 expression assessed by CPS was not independently associated with survival or recurrence outcomes in surgically treated OSCC at the prespecified cutoff of CPS ≥ 10, nor at CPS ≥ 20. An isolated overall survival association at CPS ≥ 1 was driven by a very small PD-L1-negative reference group and should not be regarded as robust. Taken together, these data argue against a reliable standalone prognostic value of PD-L1 in the early postoperative period while the limited observation time does not permit conclusions on late recurrence or long-term survival.

**Supplementary Information:**

The online version contains supplementary material available at https://doi.org/10.1007/s10006-026-01628-3.

## Introduction

For a long time, the immune system has been recognized as an essential determinant of cancer progression [1, 2]. In oral squamous cell carcinoma (OSCC), this role is particularly pronounced: tumors arise in immunologically predisposed mucosal environments in the oral cavity, and are hence often shaped by chronic inflammatory exposures such as tobacco and alcohol, and can further be driven by viral oncogenesis [3, 4]. Yet, despite this inherently immunogenic setting, outcome assessment in clinical reality hitherto relies heavily on anatomic descriptors of tumor extent and nodal spread.

The programmed death-1 (PD-1)/programmed death-ligand 1 (PD-L1) pathway has recently emerged as a central mechanism by which OSCC evades immune surveillance. Therapeutic blocking of this axis has been described to translate into meaningful survival benefits, thereby alluding to immune checkpoint inhibition as a cornerstone of modern OSCC treatment [5, 6]. Consequently, PD-L1 expression has entered the armamentarium of clinical diagnostics.

Biologically, PD-L1 expression is not a static tumor characteristic but rather reflects a finely tuned equilibrium between tumor-intrinsic signaling pathways and the surrounding immune microenvironment. PD-L1 positivity may indicate effective immune evasion by aggressive tumors, but it may also mark an immune-reactive microenvironment with ongoing antitumor responses (i.e., inflammatory response) [7]. This biological ambivalence is mirrored in the literature: studies investigating the prognostic relevance of PD-L1 in OSCC report conflicting results, ranging from adverse survival associations to neutral or even favorable outcomes [SC^8^, ^9^]. As a consequence of this, only a subset of patients benefit from treatments targeting the PD-1/PD-L1 axis despite positive expression.

A critical gap remains between the biological relevance of PD-L1 and its practical utility as a prognostic marker in OSCC treatments and care. In daily clinical practice, multidisciplinary tumor boards are increasingly confronted with PD-L1 results in OSCC patients; yet there is little evidence to guide how this information should be interpreted in relation to overall survival, disease-free survival or risk of tumor disease (e.g., locoregional, distant, secondary carcinoma) [10]. Whether PD-L1 expression conveys independent prognostic information or remains largely neutral in surgically treated cohorts remains insufficiently adressed.

In light of this, we herein aimed to investigate the prognostic significance of PD-L1 expression in a large retrospective cohort of 156 patients with surgically treated OSCC in a single center. By integrating detailed clinicopathologic data with standardized outcome analyses, we add evidence on the relationship between PD-L1 status and survival and recurrence endpoints, to ultimately aid position PD-L1 within the broader framework of established prognostic factors in contemporary OSCC management.

## Material and methods

### Study setup

This retrospective cohort study included consecutive patients treated for OSCC at the Department of Oral and Maxillofacial Surgery, Charité-Universitätsmedizin Berlin, Germany. The institutional electronic medical records and pathology reports were reviewed to identify eligible cases and extract clinicopathologic variables, treatment details, and outcome data. The study was performed in accordance with applicable regulations and the Declaration of Helsinki and received approval from the local ethics committee (EA2/077/20).

### Patient selection

All patients with primary OSCC who underwent curative-intent surgical resection between December 2015 and April 2023 were screened. Inclusion required histologically confirmed oral squamous cell carcinoma and documented follow-up information.

Patients were excluded if any of the following applied: incomplete resection (R1/R2), history of prior radiotherapy to the head and neck region, recurrent or second primary carcinoma at baseline, or insufficient clinical/pathologic documentation for endpoint analysis.

### Data collection and variables

Demographic and baseline clinical variables included age, sex, risk factors, tumor localisation, tumor laterality, and clinical staging parameters (cT, cN, cM, cUICC). Surgical data comprised date of resection, resection status, resection margins, type of neck dissection (i.e., SND vs. MRND vs. SLNB), neck dissection laterality, and detailed documentation of primary and secondary neck levels (neck level involvement for primary and secondary carcinomas).

Pathologic variables were extracted from standardized pathology reports and included pT, pN, tumor grading (G1-G3), lymphovascular invasion, perineural invasion, depth of invasion, lymph node yield, extracapsular extension (ECE), p16 status, PD-L1 status as measured by combined positive score (CPS).

Treatment-related variables comprised adjuvant therapy, tumor radiation including dosage, and radiation of surrounding lymphatic tissues including dosage, reflecting postoperative radiotherapy and/or chemoradiotherapy as determined by the multidisciplinary tumor board.

### PD-L1 immunohistochemistry and classification

PD-L1 expression was assessed by immunohistochemistry on formalin-fixed paraffin-embedded (FFPE) tumor specimens obtained at the time of primary surgery. Staining was performed in the institutional pathology laboratory according to standardized procedures. PD-L1 status was categorized using the Combined Positive Score (CPS) as reported by board-certified pathologists. For the primary analysis, tumors were classified as PD-L1 positive versus negative using a prespecified cutoff of CPS ≥ 10. This threshold was chosen a priori to maximize the biological contrast between PD-L1-high and PD-L1-low tumors. Because CPS ≥ 1 and CPS ≥ 20 are the thresholds applied in immune checkpoint inhibitor trials, both were additionally evaluated in prespecified sensitivity analyses.

### Outcomes and follow-up definitions

Follow-up was calculated from the date of primary surgery to the date of last clinical contact or death. The primary endpoint was overall survival (OS), defined as time from surgery to death from any cause. Secondary endpoints included disease-free survival (DFS), defined as the time from surgery to first documented locoregional recurrence, distant metastasis, or death (whichever occurred first), as well as locoregional control rate (LRCR), defined as the absence of tumor recurrence at the primary site or regional lymphatic basin, and neck control rate (NCR), defined as the absence of nodal recurrence within the cervical lymphatic compartments during follow-up. Recurrence events were determined from clinical records, imaging reports, and histologic confirmation where available.

### Statistical analysis

Continuous variables are summarized as mean ± standard deviation or median with interquartile range (IQR), depending on distribution; categorical variables are presented as counts and percentages. Univariable logistic regression analyses were first performed to identify factors associated with PD-L1 positivity. Variables with *p* < 0.10 in univariable analysis were subsequently entered into the multivariable logistic regression model. Time-to-event outcomes were estimated by Kaplan–Meier methodology, and differences between groups were assessed using the log-rank test. Cox proportional hazards regression was applied to evaluate predictors of OS, DFS, and LRCR, NCR. Multivariable Cox models included prespecified confounders (e.g., age, sex, stage variables, nodal disease/ECE, grading, adjuvant therapy, and subsite) and additional variables significant in univariable analyses. Variables with very low event counts (< 10 events) were not entered into multivariable models to maintain model stability. To assess whether the results depended on the chosen cutoff, all Kaplan–Meier, log-rank and Cox analyses were repeated with PD-L1 positivity defined at CPS ≥ 1 and at CPS ≥ 20, using otherwise identical model specifications. These analyses were considered supportive and were not corrected for multiplicity. All tests employed herein were two-sided, and a *p*-value of *p* ≤ 0.05 was considered statistically significant to guide conclusions. Analyses were performed using SPSS v29.0 (IBM, Armonk, NY, USA).

## Results

### Patient cohort and baseline characteristics

A total of 156 patients with primary OSCC treated with curative-intent surgical resection were included in the analysis. The cohort comprised 92 males (59.0%) and 64 females (41.0%), with a mean age of 65.1 ± 12.7 years (range: 24–84). Risk factors were documented in 100 cases (64.1%). Among these, 56 patients (56.0%) reported a history of smoking, 9 (9.0%) reported alcohol consumption alone, and 35 (35.0%) reported combined smoking and alcohol use.

**Fig. 1:**
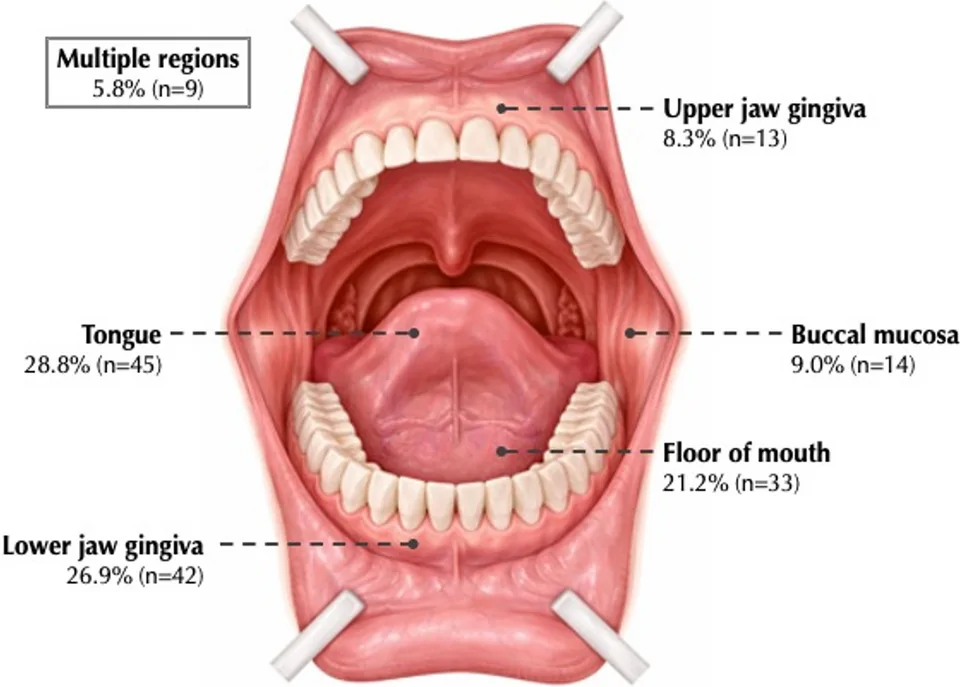
Anatomic illustration showing the primary tumor subsites in the oral cavity of the analyzed study sample. The schematic illustration was generated using AI and subsequently refined by the authors for scientific presentation

Primary tumor subsites were most common in the tongue (*n* = 45, 28.8%), mandibular gingiva (*n* = 42, 26.9%), and floor of mouth (*n* = 33, 21.2%) (Fig. 1). Clinical T stage was cT1-cT2 in 74 cases (47.4%) and cT3-cT4 in 82 cases (52.6%). UICC stage was I-II in 45 cases (28.8%) and III-IV in 111 cases (71.2%). Clinical nodal status at presentation was cN0 in 77 cases (49.4%) and cN + in 79 cases (50.6%). Pathological tumor stage was pT1 in 40 cases (25.6%), pT2 in 39 cases (25.0%), pT3 in 28 cases (17.9%), and pT4 in 49 cases (31.4%). Pathological nodal stage was pN0 in 88 cases (56.4%), pN1 in 17 cases (10.9%), pN2 in 18 cases (11.6%), and pN3 in 33 cases (21.2%). Extracapsular extension (ECE) was present in 47 cases (30.1%). Additional histopathologic features included lymphovascular invasion in 14 cases (9.0%) and perineural invasion in 68 cases (43.6%). An overview of baseline and histopathologic characteristics is provided in Table 1.

**Table 1:** Summary of patient demographics, histopathological characteristics. Categorical variables are presented as n (%), while continuous variables are reported as mean ± standard deviation (SD) [range: minimum – maximum] and median [IQR], as statistically appropriate

Total patients	156
* Male*	92 (59.0%)
* Female*	64 (41.0%)
Age [years]	65.1 (12.7) [24–84]
Disease stage (UICC)	
* I*	20 (12.8%)
* II*	25 (16.0%)
* III*	28 (17.9%)
* IVa*	77 (49.4%)
* IVb*	6 (3.8%)
Pathological T-Stage	
* T1*	40 (25.6%)
* T2*	39 (25.0%)
* T3*	28 (17.9%)
* T4a*	48 (30.8%)
* T4b*	1 (0.6%)
Pathological N-Stage	
* N0*	88 (56.4%)
* N1*	17 (10.9%)
* N2a*	4 (2.6%)
* N2b*	11 (7.1%)
* N2c*	3 (1.9%)
* N3b*	33 (21.2%)
ECE	
* positive*	47 (30.1%)
* negative*	109 (69.9%)
Lymphovascular invasion	
* positive*	14 (9.0%)
* negative*	142 (91.0%)
Perineural invasion	
* positive*	68 (43.6%)
* negative*	88 (56.4%)
Grading	
* G1*	22 (14.1%)
* G2*	108 (69.2%)
* G3*	26 (16.7%)
Depth of invasion [mm]	11.6 (10.3) [0–55]
p16 status	
* positive*	17 (10.9%)
* negative*	139 (89.1%)
PDL status	
* Tumor score (TS)*	20 [IQR: 1–70]
* Combined Tumor Cell Score (CT)*	5 [IQR: 2–10]
* Combined Positive Score (CPS)*	26 [IQR: 10–80]
Resection margin	
< *5 mm*	33 (21.2%)
≥ *5 mm*	123 (78.8%)

### PD-L1 expression patterns

A total of 120 cases (76.9%) were classified as PD-L1–positive and 36 cases (23.1%) as PD-L1-negative. In univariable logistic regression analysis, PD-L1 positivity was not significantly associated with any documented demographic or histopathological parameter (Table 2). Adjusting for confounders by including variables with *p* < 0.10 in the multivariate logistic regression model, no parameters retained independent association with PD-L1 status.

**Table 2:** Univariate binary logistic regression analyzing the association of demographic and histopathological parameters with PD-L1 positivity status. Bold values indicate statistical significance

	PD-L1 Positivity OR [95%CI]	*p value*
*Demographic parameters*		
Age	**1.000** [0.958–1.04]	0.998
Sex (Female vs. Male)	**2.127** [0.943–4.797]	0.069
Risk factors	**2.219** [0.890–5.534]	0.087
Smoking	**1.037** [0.471–2.280]	0.929
Alcohol	**2.933** [0.741–11.606]	0.125
Smoking + Alcohol	**1.500** [0.634–3.547]	0.356
*Histopathological parameters*		
pT1 (Reference)	/	/
pT2	**0.707** [0.234–2.134]	0.539
pT3	**0.636** [0.195–2.075]	0.454
pT4	**0.587** [0.209–1.651]	0.313
CLNM (pN + vs. pN0)	**1.107** [0.521–2.354]	0.791
Lymphovascular invasion (pLV)	**0.503** [0.157–1.609]	0.247
Perineural invasion (pPn)	**1.107** [0.521–2.354]	0.791
p16 Status	**0.689** [0.225–2.105]	0.513
Extracapsular extension (ECE)	**1.390** [0.596–3.242]	0.446
Grading (G1-G2 vs. G3)	**0.618** [0.243–1.568]	0.311

### Treatment characteristics

Neck management was performed in the majority of patients and consisted predominantly of selective neck dissection (SND) in 95 cases (60.9%), followed by modified radical neck dissection (MRND) in 49 cases (31.4%) and sentinel lymph node biopsy (SLNB) in 11 cases (7.1%). Complete tumor resection (R0) was achieved in all 156 patients (100.0%). Resection margins were ≥ 5 mm in 123 cases (78.8%) and < 5 mm in 33 cases (21.2%).

**Fig. 2:**
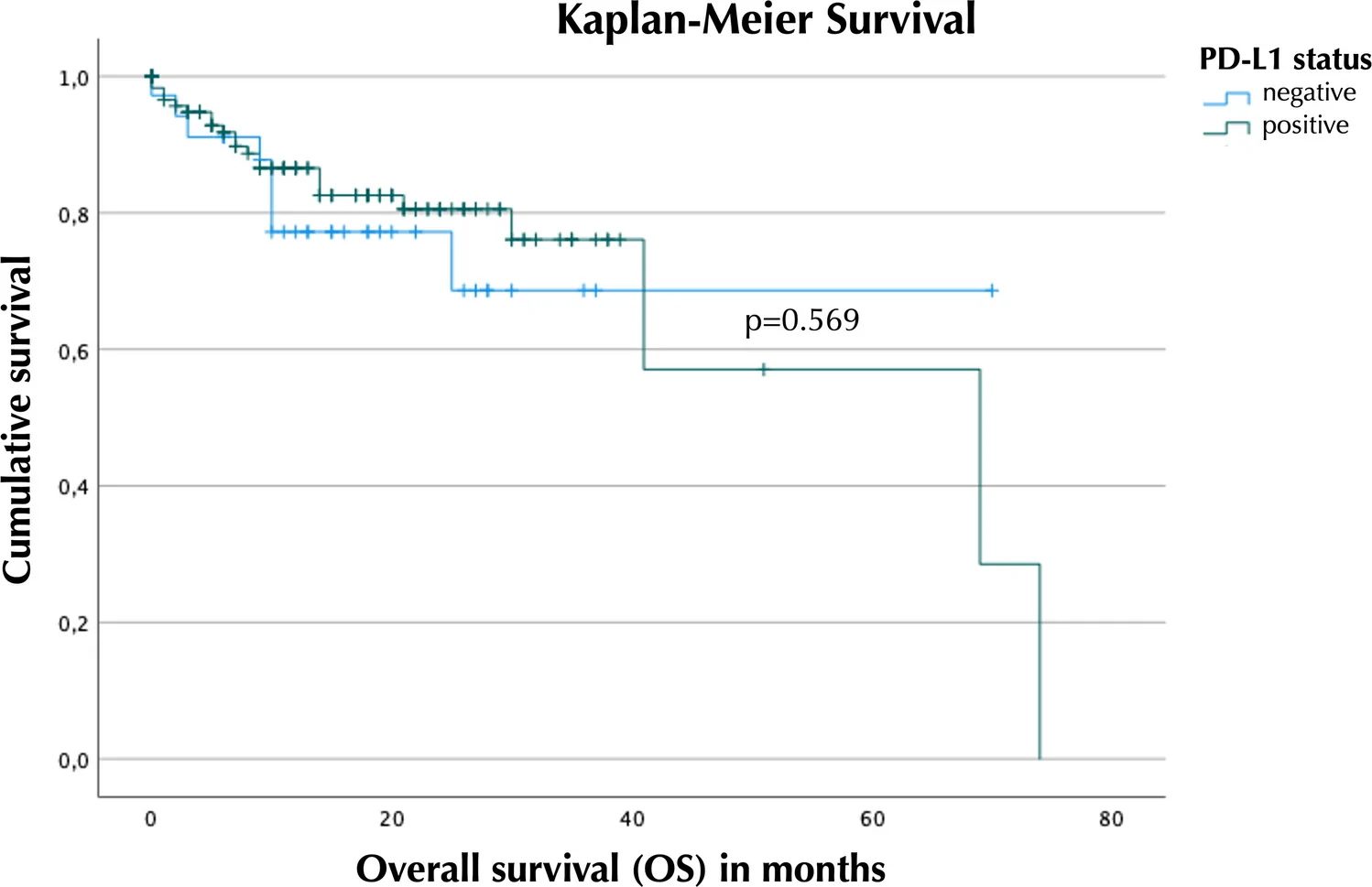
Kaplan-Meier survival curve for overall survival (OS) comparing patients with PD-L1-positiveand PD-L1-negative tumors

**Fig. 3:**
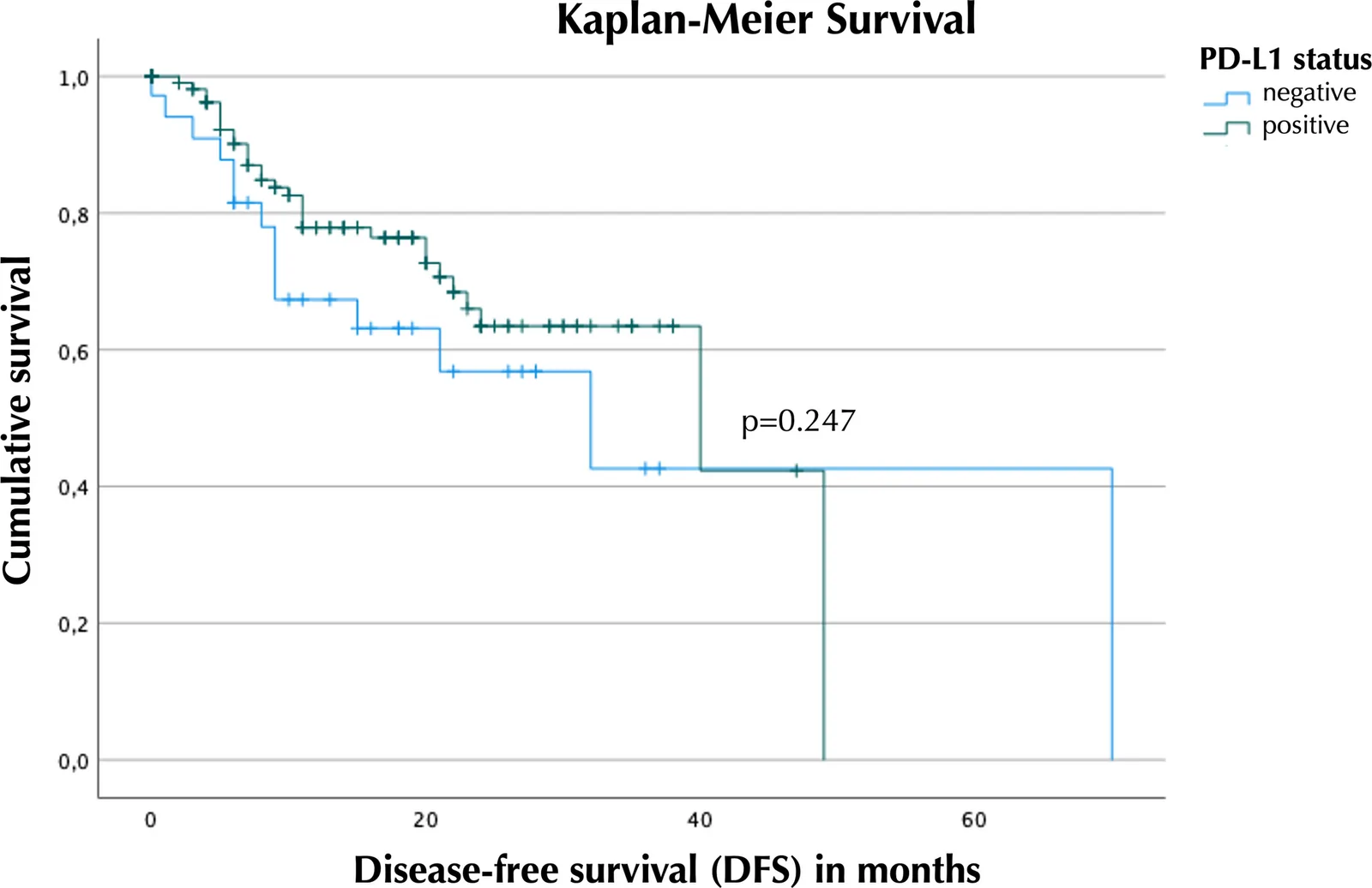
Kaplan-Meier survival curve for disease-free survival (DFS) comparing patients with PD-L1-positiveand PD-L1-negative tumors

The mean lymph node yield was 38.2 ± 23.8 lymph nodes [range: 1–156]. Adjuvant treatment was administered in 63 patients (40.4%), including postoperative radiotherapy in 37 patients (23.7%) and postoperative chemoradiotherapy in 26 patients (16.7%). The mean radiation dose to the primary tumor bed was 59.0 ± 7.4 Gy [range: 19.8–72.0], while the mean dose to the nodal drainage regions was 58.4 ± 6.8 Gy [range: 50.0–72.0] (Table 1; Figs. 2 and 3).

### Follow-up and oncologic events

The mean follow-up was 15.7 (12.5) months [range: 0–70] with a median of 14.0 months. Only a minority of patients contributed observation time beyond two years: for overall survival, 40 patients remained at risk at 24 months and 11 at 36 months (disease-free survival: 34 and 9; locoregional control: 36 and 10; neck control: 39 and 11). During follow-up, a total of 30 (19.2%) patients died. Any recurrence was observed in 52 (33.3%) cases, with locoregional recurrence in 27 (17.3%), distant metastasis in 15 (9.6%) cases, CLNM in 5 (3.2%) and secondary carcinoma in 13 (8.3%) cases. PD-L1 expression status was not associated on a statistically significant level with any of these recurrence patterns or metastatic outcomes. At 3 years, Kaplan–Meier estimates were: OS 73.9% and DFS 57.8%, with LRCR 76.9% and NCR 92.5% for the overall cohort.

### Survival outcomes by PD-L1 status

**Fig. 4:**
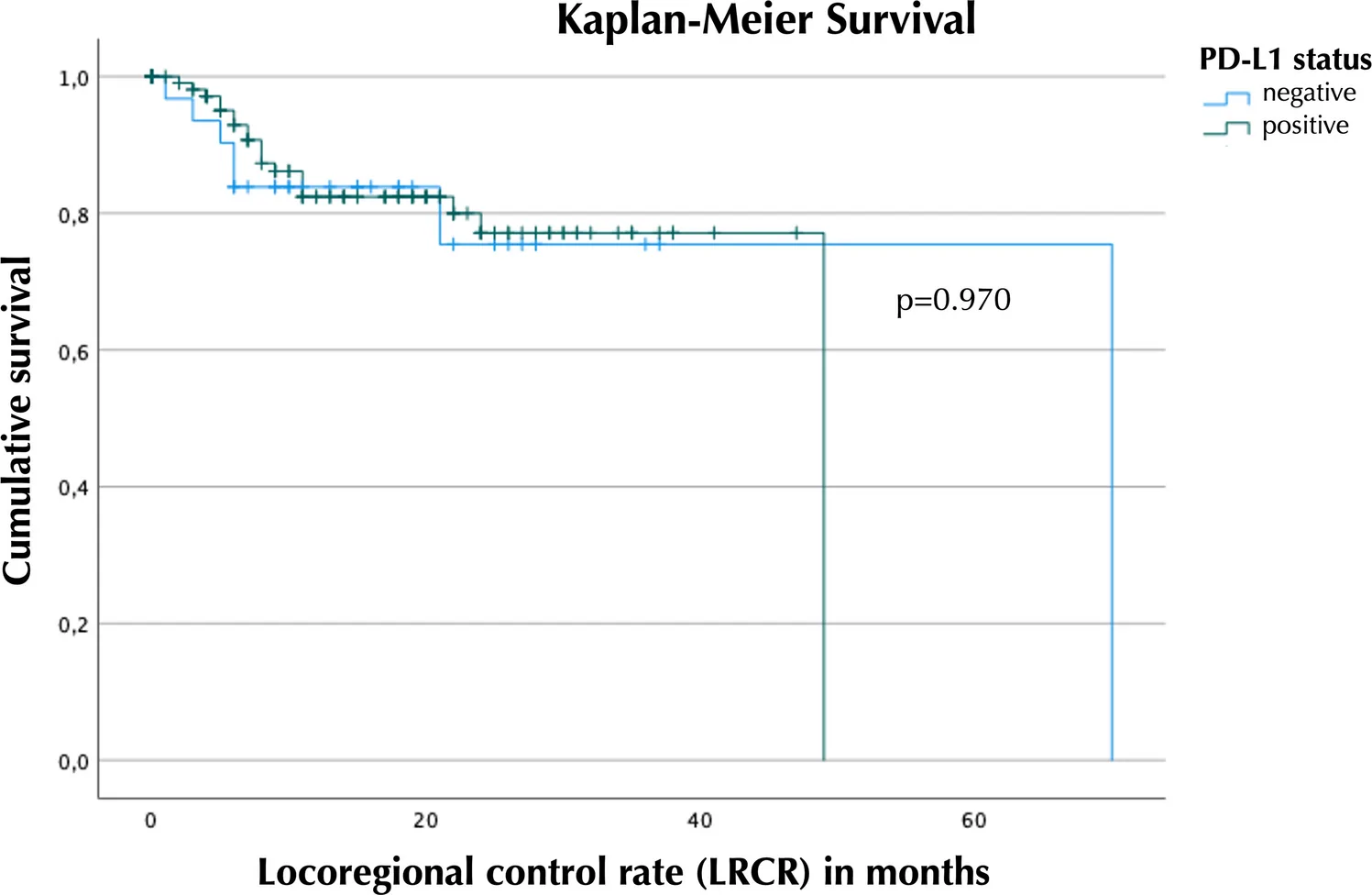
Kaplan-Meier survival curve for locoregional control rate (LRCR) comparing patients with PD-L1-positive and PD-L1-negative tumors

**Fig. 5:**
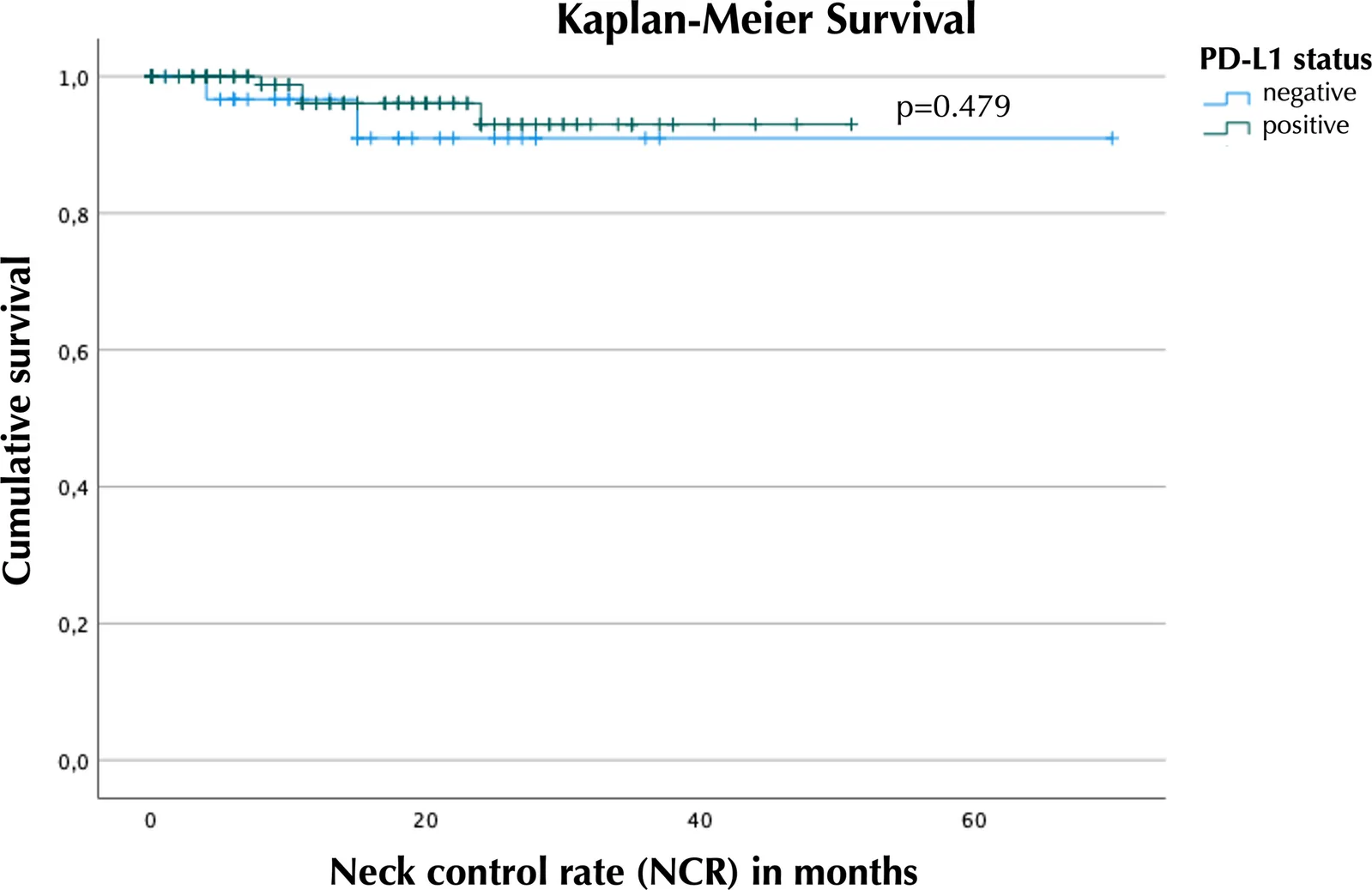
Kaplan-Meier survival curve for neck control rate (NCR) comparing patients with PD-L1-positiveand PD-L1-negative tumors

Cox regression analysis demonstrated similar OS for PD-L1-positive compared with PD-L1-negative tumors with HR = 0.791 [95% CI: 0.350–1.788] and *p* = 0.572. The estimated 3-year OS was 76.1% in the PD-L1-positive group versus 68.7% in the PD-L1-negative group, with *p* = 0.569 (Fig. 4). Similarly, DFS was comparable between groups with HR = 0.683 [95% CI: 0.355–1.314] and *p* = 0.254 and an estimated 3-year DFS of 63.5% in the PD-L1-positive group versus 42.6% in the PD-L1-negative group, with *p* = 0.247 (Fig. 5). LRCR yet again was comparable between groups with HR = 0.982 [95% CI: 0.389–2.478] and *p* = 0.970 and an estimated 3-year LRCR of 77.1% in the PD-L1-positive group versus 75.5% in the PD-L1-negative group, with *p* = 0.970. NCR was comparable between groups with HR = 0.547 [95% CI: 0.100–2.990] and *p* = 0.487 and an estimated 3-year NCR of 93.0% in the PD-L1-positive group versus 91.0% in the PD-L1-negative group, with *p* = 0.479.

When PD-L1 CPS was analyzed as a continuous variable, no significant association with survival outcomes was observed, including OS with HR = 1.003 [95% CI: 0.994–1.013] and *p* = 0.504, DFS with HR = 1.003 [95% CI: 0.995–1.012] and *p* = 0.421, LRCR with HR = 1.007 [95% CI: 0.996–1.018] and *p* = 0.118, and NCR with HR = 1.004 [95% CI: 0.982–1.026] and *p* = 0.755. Multivariable Cox regression for OS, DFS, LRCR and NCR are shown in Tables 3, 4, 5, 6.

**Table 3:** Multivariable Cox regression analysis of demographic, and histopathological parameters associated with overall survival (OS). Hazard ratios (HR) with 95% confidence intervals (CI) and *p*-values are reported. Bold values indicate statistical significance

	*P* value	Hazard Ratio	95% CI Lower boundary	95% CI Upper boundary
PDL (CPS Value)	0.563	1.004	0.991	1.016
Risk factors	0.649	1.242	0.488	3.157
Sex	0.390	1.469	0.611	3.531
pT1 (Reference)	0.405			
pT2	0.994	1.006	0.225	4.494
pT3	0.203	2.511	0.609	10.354
pT4	0.276	2.095	0.554	7.926
CLNM (pN + vs. pN0)	0.288	2.573	0.450	14.723
Lymphovascular invasion	0.053	3.377	0.984	11.591
Perineural invasion	0.297	2.423	0.459	12.779
p16 status	0.398	1.630	0.525	5.065
Extracapsular extension (ECE)	0.410	0.670	0.258	1.738
Grading (G1-G2 vs. G3)	0.068	2.340	0.939	5.834

**Table 4:** Multivariable Cox regression analysis of demographic, and histopathological parameters associated with disease-free survival (DFS). Hazard ratios (HR) with 95% confidence intervals (CI) and *p*-values are reported. Bold values indicate statistical significance

	*P* value	Hazard Ratio	95% CI Lower boundary	95% CI Upper boundary
PDL (CPS Value)	0.927	1.000	0.990	1.011
Risk factors	0.289	0.673	0.324	1.399
Sex	0.114	0.536	0.247	1.163
pT1 (Reference)	0.618			
pT2	0.914	0.945	0.341	2.622
pT3	0.797	1.167	0.360	3.782
pT4	0.341	1.629	0.597	4.441
CLNM (pN + vs. pN0)	0.946	1.043	0.304	3.585
Lymphovascular invasion	0.528	1.456	0.454	4.674
Perineural invasion	0.066	3.015	0.930	9.768
p16 status	0.731	0.807	0.237	2.741
Extracapsular extension (ECE)	0.109	1.871	0.870	4.023
Grading (G1-G2 vs. G3)	0.616	1.222	0.558	2.680

**Table 5:** Multivariable Cox regression analysis of demographic, and histopathological parameters associated with the locoregional control rate (LRCR). Hazard ratios (HR) with 95% confidence intervals (CI) and *p*-values are reported. Bold values indicate statistical significance

	*P* value	Hazard Ratio	95% CI Lower boundary	95% CI Upper boundary
PDL (CPS Value)	0.434	1.005	0.992	1.018
Risk factors	0.526	0.743	0.297	1.858
Sex	0.130	0.446	0.157	1.270
pT1 (Reference)	0.547			
pT2	0.997	1.003	0.241	4.177
pT3	0.832	1.192	0.234	6.067
pT4	0.293	2.119	0.522	8.595
CLNM (pN + vs. pN0)	0.814	1.205	0.255	5.690
Lymphovascular invasion	0.839	1.182	0.236	5.911
Perineural invasion	0.333	2.052	0.478	8.807
p16 status	0.499	0.493	0.064	3.827
Extracapsular extension (ECE)	0.259	1.747	0.663	4.604
Grading (G1-G2 vs. G3)	0.902	0.935	0.321	2.726

**Table 6:** Multivariable Cox regression analysis of demographic, and histopathological parameters associated with the neck-control rate (NCR). Hazard ratios (HR) with 95% confidence intervals (CI) and *p*-values are reported. Bold values indicate statistical significance

	*P* value	Hazard Ratio	95% CI Lower boundary	95% CI Upper boundary
PDL (CPS Value)	0.453	1.010	0.984	1.038
Risk factors	0.401	3.400	0.196	59.115
Sex	0.359	0.337	0.033	3.449
pT1 (Reference)	0.745			
pT2	0.977	0.971	0.134	7.059
pT3	0.980	0.000	0.000	
pT4	0.325	0.196	0.008	5.006
CLNM (pN + vs. pN0)	0.463	0.320	0.015	6.708
Lymphovascular invasion	0.139	12.353	0.444	343.863
Perineural invasion	0.071	11.968	0.809	176.996
p16 status	0.603	1.982	0.151	26.095
Extracapsular extension (ECE)	0.531	2.056	0.216	19.584
Grading (G1-G2 vs. G3)	0.460	2.609	0.205	33.174

Stage-stratified analysis revealed no association between PD-L1 status and survival outcomes in either early-stage (UICC I-II) or advanced-stage disease (UICC III-IV), including overall survival (*p* = 0.486 and *p* = 0.585), disease-free survival (*p* = 0.256 and *p* = 0.143), locoregional control rate (*p* = 0.378 and *p* = 0.843), and neck control rate (*p* = 0.458 and *p* = 0.201).

In analyses stratified by adjuvant treatment, PD-L1 status was not associated with OS in patients without or with adjuvant therapy, with *p* = 0.124 and *p* = 0.625, respectively. For DFS, PD-L1–positive tumors were associated with longer DFS among patients who did not receive adjuvant therapy (*p* = 0.027), whereas no difference was observed in patients treated with adjuvant therapy (*p* = 0.874). PD-L1 status was not associated with LRCR (*p* = 0.987 and *p* = 0.552) or NCR (*p* = 0.684 and *p* = 0.317) in either treatment subgroup.

### Sensitivity analyses across CPS thresholds

Applying the alternative cutoffs, 145 tumors (92.9%) were classified as PD-L1-positive at CPS ≥ 1 and 97 (62.2%) at CPS ≥ 20, compared with 120 (76.9%) at the prespecified cutoff of CPS ≥ 10. At CPS ≥ 20, PD-L1 status was not associated with OS (HR = 1.158 [95% CI: 0.537–2.497]; *p* = 0.707), DFS (HR = 1.207 [95% CI: 0.643–2.265]; *p* = 0.558), LRCR (HR = 1.366 [95% CI: 0.589–3.169]; *p* = 0.468) or NCR (HR = 0.611 [95% CI: 0.123–3.030]; *p* = 0.547), thus reproducing the primary analysis. At CPS ≥ 1, PD-L1 positivity was associated with superior OS (HR = 0.280 [95% CI: 0.106–0.745]; *p* = 0.011; log-rank *p* = 0.006), with a 3-year OS of 76.2% versus 46.8%, whereas DFS (HR = 0.528 [95% CI: 0.187–1.490]; *p* = 0.228) and LRCR (HR = 0.693 [95% CI: 0.162–2.961]; *p* = 0.621) remained non-significant; NCR was not estimable at this threshold because no neck recurrence occurred among PD-L1-negative patients. This threshold defines a PD-L1-negative reference group of only 11 patients, all with a CPS of 0, in whom 5 deaths occurred, all within the first 10 months of follow-up. Multivariable modelling at this threshold was not interpretable given 11 patients in the reference group and 27 events across 12 covariates, and is therefore not reported. The exploratory DFS difference observed among adjuvant-treatment-naïve patients at CPS ≥ 10 (*p* = 0.027) was not reproduced at either alternative threshold (CPS ≥ 1: *p* = 0.092; CPS ≥ 20: *p* = 0.552). Effect estimates across all three thresholds are summarized in Supplementary Table 1.

## Discussion

Using a CPS threshold of ≥ 10, a total of 76.9% of tumors were considered PD-L1-positive, a rate at the higher end of published estimates [11, 12]. Unlike the TPS, which quantifies only tumor cell staining, the CPS incorporates immune cells alongside tumor cells, thereby naturally yielding higher positivity rates in comparison [13]. The sensitivity of the PD-L1-positivity rate to the chosen threshold is well established in the literature with studies reporting rates ranging from nearly 90% at CPS ≥ 1 to under 45% at CPS ≥ 20 within the same cohort [14]. To ensure that the present findings were not an artifact of the selected cutoff (i.e., CPS ≥ 10, the analyses presented herein were repeated at CPS ≥ 1 and CPS ≥ 20. At CPS ≥ 20 the null findings were reproduced across all four endpoints. At CPS ≥ 1, by contrast, PD-L1 positivity was associated with superior OS. This apparent discrepancy is best explained by the composition of the comparison groups rather than by a biological threshold effect: at CPS ≥ 1 the PD-L1-negative reference group comprises 11 patients with a CPS of 0, in whom five deaths occurred within the first ten months, and no neck recurrence occurred at all, rendering that endpoint non-estimable. An effect estimate resting on five early events in eleven patients is statistically fragile and should not be read as evidence of a prognostic role. The wider implication shown hereby is that reported associations between PD-L1 and outcome in OSCC in the literature may depend substantially on how few patients fall into the negative reference category at a given cutoff, which offers one concrete explanation for the heterogeneity of published findings.

The absence of a prognostic association across all four endpoints, whether PD-L1 was analyzed as a binary or as a continuous variable (i.e., CPS score), aligns closely with recent literature. In their systematic review and meta-analysis, Nocini et al. reported a pooled HR for OS of 0.97 and for DFS of 0.83, both non-significant, while Troiano et al. similarly found no correlation between tumor-cell PD-L1 expression and prognosis with a pooled HR of 0.60 for OS and 0.62 for DFS [13, 15]. A discordant meta-analysis by Lenouvel et al. reported worse disease-specific survival and DFS for high PD-L1 expressors. Yet, this finding needs to be interpreted in light of an absence of standardized scoring across included studies [16]. An overview of systematic reviews by Al-Azzawi et al. [6] ultimately concluded that current evidence does not support PD-L1 as a reliable prognostic marker for OS in OSCC, a conclusion the present data reinforce in a well-defined surgically treated cohort. In this context, it should be emphasized that the present analysis captures the early postoperative interval. In a surgically treated OSCC cohort of 760 patients, only 24% of all recurrences had become manifest at 12 months and 50% at 24 months, with the remaining half distributed over the subsequent years and the latest event recorded after 135 months [17]. With a mean follow-up of 15.7 months, the present cohort therefore covers only a fraction of the interval during which recurrences arise, and the absence of an association is best read as the absence of an early prognostic signal rather than as evidence that PD-L1 carries no prognostic information over the full disease course.

A potential biological explanation for these inconsistent findings is that “PD-L1 positivity” can aggregate biologically distinct tumor states. In some tumors, PD-L1 expression may be driven predominantly by tumor-cell-intrinsic oncogenic programs and reflect immune escape, whereas in others it is triggered by IFN-γ within a T-cell-inflamed microenvironment, consistent with adaptive immune resistance. When these distinct “etiologies” are collapsed into a single ‘PD-L1-positive’ category, the net prognostic association may be obscured at the cohort level. This interpretation is supported by studies showing that the prognostic relevance of PD-L1 in HNSCC/OSCC depends on immune context, particularly the density of co-infiltrating CD8 + tumor-infiltrating lymphocytes, rather than PD-L1 status alone [18, 19].

The nominally significant improvement in DFS among PD-L1-positive patients not having received adjuvant treatment warrants cautious interpretation given its exploratory nature in this study. A plausible explanation is that in purely surgically managed patients, IFN-γ-driven PD-L1 positivity reflects a more immune-active microenvironment that delays recurrence, a signal potentially obscured in adjuvant-treated patients by the complex immunomodulatory effects inherent to radiotherapy. Consistent with this hypothesis, a meta-analysis by Polesel et al. in oropharyngeal SCC found a significant survival benefit for PD-L1-positive patients treated with upfront surgery (sHR = 0.34, 95% CI 0.18–0.65), whereas no such association was observed in patients receiving primary chemoradiotherapy, suggesting a prognostic relevance which could depend on treatment-modality. Whether a comparable interaction exists in the OSCC patients investigated herein, remains largely speculative and warrants prospective investigation.

Indirectly, these results also have a direct clinical implication: PD-L1’s validated utility in OSCC lies in predicting response to immune checkpoint inhibition, not in stratifying prognosis after curative surgery. KEYNOTE-048 established this predictive role, demonstrating survival benefits for pembrolizumab over cetuximab-chemotherapy in the PD-L1-positive populations, both for CPS ≥ 1 and CPS ≥ 20, in the recurrent/metastatic setting [20, 21]. Extrapolating this predictive value to the prognostic domain in R0-resected patients is an inferential error, and tumor boards should resist drawing survival inferences from PD-L1 scores in this setting.

This study is not free of limitations: First, its retrospective single-center design naturally entails an inherent risk of selection and information bias and limits generalizability beyond surgically treated, R0-resected OSCC patients managed at a tertiary referral center. Second, the mean follow-up of 15.7 ± 12.5 months is short relative to the natural history of OSCC, with the 3-year estimates resting on comparatively few observations, and late recurrences, second primary tumors and long-term survival cannot be meaningfully assessed. The null findings reported here should therefore be understood as applying to the early postoperative period, and a prognostic effect of PD-L1 emerging with longer observation cannot be excluded. Third, the number of events was low for some endpoints, particularly neck recurrence, which reduces statistical power, results in wide confidence intervals, and increases the risk that modest prognostic effects remained undetected. Fourth, PD-L1 assessment was based on routine pathology reporting without central slide re-review or formal interobserver analysis, and heterogeneity of CPS-based classification is well-known to be prone to specimen selection, intratumoral heterogeneity, analytical conditions, and the applied cutoff. This could be demonstrated with the sensitivity analyses conducted at CPS ≥ 1 and CPS ≥ 20, at which the PD-L1-negative group in our cohort becomes too small to support stable effect estimates. Comparability with TPS-based or immune-cell-specific scoring approaches nonetheless remains limited. Finally, no systematic quantification of CD8 + TILs or broader immune microenvironment features was available, precluding a context-dependent interpretation of PD-L1 expression. These limitations may have attenuated the detectable prognostic signal of PD-L1 and should be considered when interpreting the present null findings. Future studies ought to be designed with these limitations in mind; particularly by incorporating prospective multicenter cohorts, longer follow-ups, standardized scoring, and concurrent immune microenvironment profiling.

## Conclusion

In this large single-center cohort of surgically treated OSCC patients with curative intent, PD-L1 expression assessed by CPS was not independently associated with OS, DFS, LRCR, or NCR within a mean follow-up of 15.7 months. This was equally true at the higher threshold of CPS ≥ 20, whereas the OS association seen at CPS ≥ 1 rested on an 11-patient reference group and is not considered robust. Although PD-L1-positive tumors were associated with improved disease-free survival in the subgroup of adjuvant-treatment-naïve patients, this finding was exploratory and should be interpreted with caution. Overall, these findings suggest that PD-L1 has limited standalone prognostic value in the early postoperative course of the curative surgical setting and should be interpreted cautiously outside its established role as a predictive biomarker for immune checkpoint inhibition. Future prospective studies with extended follow-up, integrating standardized PD-L1 assessment with advanced immune microenvironment profiling, are warranted to better understand its prognostic relevance in OSCC.

## Supplementary Information

Below is the link to the electronic supplementary material.Supplementary file1 (DOCX 92 KB)

## Data Availability

No datasets were generated or analysed during the current study.
