# Supplementary material for: Programmed Death-Ligand 1 (PD-L1) expression and clinical outcomes in oral squamous cell carcinoma: evidence from a large clinical cohort
Source: Oral Maxillofac Surg. 2026 Aug 29;30(1):144. doi: 10.1007/s10006-026-01628-3 (PMC13525057; doi:10.1007/s10006-026-01628-3)
Supplement: Supplementary file 1 [file 10006_2026_1628_MOESM1_ESM.docx]

**Programmed Death-Ligand 1 (PD-L1) Expression and Clinical Outcomes in Oral Squamous Cell Carcinoma: Evidence from a Large Clinical Cohort**

Michael **Alfertshofer** MD^1,2^, Maximilian **Richter** MD DMD^1^, Konrad **Klinghammer** MD^3^, Christian **Doll** MD DMD^1^, Steffen **Koerdt** MD DMD^1^, Max **Heiland** MD DMD^1^, Friedrich **Mrosk** MD DMD^1^

^1^ Charité – Universitätsmedizin Berlin, corporate member of Freie Universität Berlin, and Humboldt-Universität zu Berlin, Department of Oral and Maxillofacial Surgery, Berlin, Germany

^2^ Department of Plastic and Hand Surgery, Technical University Munich, Munich, Germany

^3^ Charité – Universitätsmedizin Berlin, Comprehensive Cancer Center, corporate member of Freie Universität Berlin, and Humboldt-Universität zu Berlin, Division of Hematology, Oncology and Tumorimmunololgy, Berlin, Germany

*Keywords*: Head and neck squamous cell carcinoma, PD-L1 expression, prognostic biomarkers, overall survival

*Running title*: PD-L1 Expression and Outcomes in OSCC

*Author disclosure*: The authors declared no potential conflicts of interest with respect to the research, authorship, and publication of this article.

*Funding*: The authors received no financial support for the research, authorship, and publication of this article. The products utilized in this study were donated by the injectors for the purposes of this study.

Corresponding author:

**Michael Alfertshofer MD**

Department of Oral and Maxillofacial Surgery

Charité – Universitätsmedizin Berlin

Berlin

Germany

E-Mail: Michael-georg.alfertshofer@charite.de

*Supplementary Table 1*: Sensitivity analysis of the association between PD-L1 status and oncologic endpoints across three CPS thresholds: CPS ≥1, CPS ≥10 and CPS ≥20. At CPS ≥1 the PD-L1-negative reference group comprises 11 patients; no neck recurrence occurred in this group, so the hazard ratio for NCR is not estimable and the log-rank p-value is given instead.

| **Endpoint** | **CPS ≥1** | **CPS ≥10 (primary)** | **CPS ≥20** |
| --- | --- | --- | --- |
| PD-L1-positive, n (%) | 145 (92.9%) | 120 (76.9%) | 97 (62.2%) |
| Overall survival (OS) | HR=0.280; 95% CI 0.106–0.745; p=0.011 | HR=0.791; 95% CI 0.350–1.788; p=0.572 | HR=1.158; 95% CI 0.537–2.497; p=0.707 |
| Disease-free survival (DFS) | HR=0.528; 95% CI 0.187–1.490; p=0.228 | HR=0.683; 95% CI 0.355–1.314; p=0.254 | HR=1.207; 95% CI 0.643–2.265; p=0.558 |
| Locoregional control rate (LRCR) | HR=0.693; 95% CI 0.162–2.961; p=0.621 | HR=0.982; 95% CI 0.389–2.478; p=0.970 | HR=1.366; 95% CI 0.589–3.169; p=0.468 |
| Neck control rate (NCR) | not estimable; log-rank p=0.588 | HR=0.547; 95% CI 0.100–2.990; p=0.487 | HR=0.611; 95% CI 0.123–3.030; p=0.547 |
